# On the Alteration of the Taste in Paralysis of the Facial Nerve

**Published:** 1853-05

**Authors:** 


ART. XVII.—*On the Alteration of the Taste in Paralysis of the Facial Nerve.* By M. CLAUDE BERNARD, Lecturer on Physiology at the College of France. Translated from the French by HOWELL L. THOMAS, M. D.

In this memoir, M. Bernard cites examples going to establish a connection frequently remarked by observers between the facial nerve and the sense of taste. The author attempts a physiological explanation of the phenomenon, which is not "a complete abolition of gustation, but a simple diminution and sort of perversion in this faculty."

"It is to be observed that this sensorial modification is independent of the tactile sensibility of the mucous surface of the tongue which does not suffer any diminution. In relation to its seat, the alteration of taste, limited to the anterior two thirds of the tongue, only manifests itself upon the half of the tongue which corresponds to the hemiplegic side. And as a main character, it follows exactly the march of the other symptoms of the facial paralysis, since it appears and disappears *pari passu* with them. These symptoms will always serve to distinguish the alteration of taste due to the influence of the facial nerve from that which might depend upon a concomitant lesion of the fifth pair. Thus it is unquestionable that it is in a lesion of the facial nerve that we must search for the anatomical reason for the alteration of taste of which we are now treating. Anatomically speaking, there is but one branch of the facial nerve which can be invoked for this explanation; this is the chorda tympani which, after traversing its singular course through the internal ear, unites and confounds itself with the lingual nerve. The experiments upon living animals are demonstrative in this respect. If the facial nerve of a dog be cut above the origin of the chorda tympani, or if this nervlous filament is isolated by seizing it in its passage through the internal ear by means of a small crochet introduced by the external meatus, you can determine with the animal the same gustative diminution, with the characters above described.

Without reconsidering the experiments of this nature which we have reported at length elsewhere, we will limit ourselves to referring to their results in regard to the pathological phenomena observed in man. From the summary of these facts, we think we can infer with certainty, 1st, that the alteration of taste will be wanting in facial hemiplegia whenever the paralysing cause, whatever it may be, affects the facial nerve below the emergence of the chorda tymp.ıni in such a way as not to compromise the action of this nervous filament; 2d, that the sensorial symptom, on the contrary, will constantly exist when the paralysing cause is seated sufficiently high in the course of the seventh pair to destroy the function of the chorda tympani. We understand then that all the causes of facial paralysis which have their seat or point of departure in the internal ear must necessarily compromise this nervous thread and produce the alteration of taste; The observations 1, 2 and 3 furnish examples of this kind. Thus prthology and physiology accord in explaining how we may ha"e facial paralysis with or without alteration of taste, according as the chorda tympani is implicated or not. Moreover, as the tympanitic branch is free and isolated in its passage through the cavity of the tympanum, it may be cenceived that certain causes, traumatic or others, destroying or isolating this nerve, may produce only perversion of taste, without any symptom of facial paralysis. This fact may be easily proved by experiment upon the living animal."

